# The importance of maternal pregnancy vitamin D for offspring bone health: learnings from the MAVIDOS trial

**DOI:** 10.1177/1759720X211006979

**Published:** 2021-04-08

**Authors:** Rebecca J. Moon, Elizabeth M. Curtis, Stephen J. Woolford, Shanze Ashai, Cyrus Cooper, Nicholas C. Harvey

**Affiliations:** MRC Lifecourse Epidemiology Unit, University of Southampton, Southampton General Hospital, Tremona Road, Southampton, Hampshire SO16 6YD, UK; MRC Lifecourse Epidemiology Unit, University of Southampton, Southampton General Hospital, Southampton, Hampshire, UK; MRC Lifecourse Epidemiology Unit, University of Southampton, Southampton General Hospital, Southampton, Hampshire, UK; MRC Lifecourse Epidemiology Unit, University of Southampton, Southampton General Hospital, Southampton, Hampshire, UK; MRC Lifecourse Epidemiology Unit, University of Southampton, Southampton General Hospital, Southampton, Hampshire, UK; NIHR Southampton Biomedical Research Centre, University of Southampton and University Hospital Southampton NHS Foundation Trust, Southampton, UK; NIHR Biomedical Research Centre, University of Oxford, Oxford, UK; MRC Lifecourse Epidemiology Unit, University of Southampton, Southampton General Hospital, Southampton, Hampshire, UK; NIHR Southampton Biomedical Research Centre, University of Southampton and University Hospital Southampton NHS Foundation Trust, Southampton, UK

**Keywords:** bone mineral density, bone turnover markers, cholecalciferol, epigenetics, Maternal Vitamin D Osteoporosis Study, osteoporosis, pregnancy

## Abstract

Optimisation of skeletal mineralisation in childhood is important to reduce childhood fracture and the long-term risk of osteoporosis and fracture in later life. One approach to achieving this is antenatal vitamin D supplementation. The Maternal Vitamin D Osteoporosis Study is a randomised placebo-controlled trial, the aim of which was to assess the effect of antenatal vitamin D supplementation (1000 IU/day cholecalciferol) on offspring bone mass at birth. The study has since extended the follow up into childhood and diversified to assess demographic, lifestyle and genetic factors that determine the biochemical response to antenatal vitamin D supplementation, and to understand the mechanisms underpinning the effects of vitamin D supplementation on offspring bone development, including epigenetics. The demonstration of positive effects of maternal pregnancy vitamin D supplementation on offspring bone development and the delineation of underlying biological mechanisms inform clinical care and future public-health policies.

## Introduction

Optimising childhood bone health is important. Fracture in children and adolescents is common; approximately one third of boys and one fifth of girls will sustain a fracture by the age of 18 years,^1^ with implications for loss of education and parental earnings, pain, reduced physical functioning and use of healthcare resources.^2^ The majority of fractures in children occur in those with normal bone strength due to trauma. However, reduced bone strength due to impaired bone mineralisation and alterations in bone microarchitecture do increase the propensity to fracture.^3,4^

Bone mineralisation in childhood also establishes a trajectory for adult bone health. During childhood and adolescence, the skeleton grows in both length and width, resulting in an increase in bone mass [the composite of bone mineral content (BMC) and bone size]. Although final height is reached shortly after the end of puberty, bone mineral accrual continues into the third decade, with peak bone mass (PBM) being reached in the mid to late 20s. Thereafter, bone mass declines, with an acceleration in the rate of bone loss after the menopause in women. Whilst PBM is in part genetically determined, external factors that modify an individual’s ability to achieve their genetic potential might moderate osteoporosis risk, and indeed mathematical modelling has shown that a modest increase in PBM can substantially delay the onset of osteoporosis.^5^ Osteoporotic fractures in later life are associated with increased mortality,^6^ poorer quality of life and functional decline,^7^ and are a significant cause of healthcare spending.^8^ There is an urgent need for approaches to reducing this burden, and importantly, there is increasing evidence to support targeting early life skeletal development. One such potential intervention is antenatal vitamin D supplementation. In this review, we review and discuss the findings and clinical implications of the Maternal Vitamin D Osteoporosis Study (MAVIDOS), the largest randomised placebo-controlled trial of antenatal cholecalciferol supplementation specifically aiming to address the effects on offspring musculoskeletal health,^9^ in the context of the wider evidence base.

### Foetal skeletal mineralisation

Skeletal development begins from 8 weeks to 12 weeks gestation and requires synchronisation of chondrogenesis, osteogenesis and synovial joint formation. Mineralisation of bone templates generated through intramembranous and endochondral ossification principally occurs during the third trimester when 80% of bone mineral is accreted. During pregnancy, maternal Ca^2+^ is actively transported across the placenta to the foetus, resulting in a greater plasma Ca^2+^ concentration in the foetus compared with the mother.^10^ A doubling of maternal fractional absorption of calcium through the intestine from as early as 12 weeks gestation and maintained until delivery, and to a lesser extent, resorption of the maternal skeleton during the third trimester facilitates the availability of Ca^2+^ to meet foetal demands.^11–13^ This is achieved through alterations to maternal calcitropic hormones, including an increase in parathyroid-related peptide and 1,25 dihydroxyvitamin D [1,25(OH)_2_D].^12,14^ During the final 6 weeks of gestation, calcium transfers across the placenta at a rate of 300 mg/day, and at term the average foetal skeleton will contain approximately 30 g of calcium in addition to 20 g phosphorus and 0.8 g magnesium.^11^ Limited availability of substrates for bone mineralisation, for example due to maternal diet, impaired maternal intestinal function, maternal vitamin D deficiency or impaired placental function/transfer are therefore likely to impact negatively on bone mineralisation during *in utero* life.

### Vitamin D in pregnancy

Considering the importance of maternal vitamin D to the upregulation of intestinal calcium absorption, it is not surprising that antenatal vitamin D status has been explored as a possible approach to improving offspring skeletal mineralisation.

Vitamin D is a group of fat-soluble secosteroids, of which cholecalciferol (vitamin D3) and ergocalciferol (vitamin D2) are the most common forms. Vitamin D can be obtained from dietary sources including oily fish, eggs and fortified milk, but the majority is synthesised in the skin from the action of ultraviolet-B to convert 7-dehydrocholesterol to pre-vitamin D3. This is then hydroxylated in the liver to its circulating form, 25-hydroxyvitamin D [25(OH)D]. This circulating 25(OH)D acts as a reservoir for conversion to the active metabolite, 1,25(OH)_2_D, the classical function of which is calcium and phosphate homeostasis, although other non-classical functions including in immunological, muscular and neurological functions are increasingly documented.

Serum 25(OH)D levels rather than 1,25(OH)_2_D^15^ are currently the best available biomarker of vitamin D status due to the longer half-life and tight physiological regulation of 1,25(OH)_2_D in response to Ca^2+^ homeostasis. There is great variability in the recommended thresholds to define vitamin D deficiency (usually between 25 nmol/L and 50 nmol/L),^16–18^ but risk factors for low serum 25(OH)D are well recognised. This includes seasonal variation (nadir in winter months), residing at latitudes far from the equator, reduced cutaneous vitamin D synthesis due to dark skin pigmentation, extensive skin colouring or limited time outdoors, and high adiposity, due to sequestration of vitamin D in adipose tissue.

As in other population groups, biochemically low levels of 25(OH)D are common in pregnant women. For example, in a study of predominately White women in the south of the UK, 31% had a serum 25(OH)D <50 nmol/L and 18% <25 nmol/L in late pregnancy.^19^ In a more ethnically diverse population in London, 36% women had 25(OH)D <25 nmol/L in early pregnancy.^20^ In the UK, the Department of Health currently recommends supplementation with 400 IU/day cholecalciferol throughout pregnancy and lactation,^21^ and the Institute of Medicine and the Global Consensus on Prevention and Management of Nutritional Rickets suggest supplementation with 600 IU/day during this period.^22,23^ Such an approach is of demonstrable benefit in reducing the incidence of symptomatic neonatal hypocalcaemia,^24–26^ with increasing evidence for a benefit for offspring skeletal health and birth weight,^27^ and maternal obstetric complications, such as pre-eclamspia.^28^

### Observational studies of maternal vitamin D status and offspring bone mineralisation

An ever-growing collection of observational studies have investigated the relationships between markers of maternal vitamin D status and offspring bone health, with inconsistent outcomes. Approaches have included utilising the known seasonal variation in maternal serum 25(OH)D status as an ecological marker of vitamin D status and direct measurement of blood 25(OH)D concentrations.

In 1998 a study from Korea found that infants born in winter months had both lower whole body BMC and umbilical cord serum 25(OH)D levels compared with those born in summer months.^29^ However, this contradicted the findings of a similar study by the same authors undertaken in the USA.^30^ Using the Avon Longitudinal Study of Parents and Children, Sayer and Tobias^31^ reported that in nearly 7000 mother–offspring pairs estimated maternal ultraviolet B (UVB) exposure in late pregnancy was positively associated with offspring whole-body less head (WBLH) BMC, bone area and bone mineral density (BMD) at 9 years of age. However, assessment of maternal serum 25(OH)D in a subset of this cohort subsequently did not reveal any associations with offspring bone mineralisation.^32^

Other studies using measurement of maternal or umbilical cord blood 25(OH)D have also reported inconsistent findings. However, the populations studied, including the distribution of vitamin D status, gestation at measurement of 25(OH)D, approach to defining 25(OH)D as a continuous or categorical outcome, and age and method at which bone mineralisation is quantified in the offspring, have varied considerably. For example, studies from Canada and Norway reported low maternal 25(OH)D status during pregnancy associated with reduced BMC in the neonatal period and at 14 months of age.^33–35^ In contrast, a cohort in The Gambia displayed no association between maternal 25(OH)D status and offspring whole-body BMC at birth or at several ages in the first year of life.^36^ In the latter of these cohorts, no mothers had a serum 25(OH)D <50 nmol/L, suggesting that maternal 25(OH)D levels may only negatively impact skeletal mineralisation in severe deficiency.

Similarly, findings from observational studies in later childhood are also inconsistent. In the Southampton’s Women’s Survey (SWS), a UK-based prospective cohort, which included 1030 maternal–offspring pairs, offspring born to mothers with a serum 25(OH)D <25 nmol/L in late pregnancy had lower whole-body bone area, BMC, areal BMD and lumbar spine BMC at 6 years compared with offspring born to mothers with a measurement above this level.^37^ Furthermore, positive associations between late pregnancy maternal 25(OH)D and offspring muscle strength at 4 years were observed in the same cohort.^38^ Given the importance of lean mass and muscle loading to bone mineralisation, this could represent a further mechanism for the effect of vitamin D supplementation on bone mineralisation. In another cohort from the same geographical area, maternal late pregnancy serum 25(OH)D was also associated with whole-body and lumbar spine BMC at 9 years.^19^ One study showed that at age 11 years, maternal 25(OH)D in early pregnancy, but not 28–32 weeks gestation, was associated with spine and WBLH BMD in boys, but not girls.^39^ The earlier age at commencement of puberty in girls might have been important to this observation. A study from Australia demonstrated the persistence of the relationship between maternal pregnancy vitamin D status and offspring bone mineralisation in young adulthood.^40^ In contrast, a study from The Netherlands with follow up at age 6 years did not support these findings.^41^ These inconsistent findings highlight the need for high-quality intervention studies to define the nature of these relationships.

## The MAVIDOS trial

MAVIDOS was the first intervention study designed specifically to assess the effects of antenatal vitamin D supplementation in pregnancy on offspring bone mineralisation.^42^

### Methodology

The trial recruited women in early pregnancy from three sites in the UK: University Hospital Southampton NHS Foundation Trust (latitude 50.9°N), John Radcliffe Hospital, Oxford (latitude 51.8°N) and Sheffield Hospitals NHS Trust (latitude 53.4°N). Women with a singleton pregnancy, not taking more than 400 IU/day vitamin D supplementation and, due to an ethical stipulation, with a baseline 25(OH)D between 25 nmol/L and 100 nmol/L were eligible to participate. Randomisation was to either 1000 IU/day cholecalciferol or matched placebo from 14 weeks gestation until delivery. Both participants and researchers were blinded to the randomisation. Detailed assessments of anthropometry, lifestyle, diet and blood sampling took place at 14 weeks and 34 weeks gestation (Table 1). At birth, infant anthropometric data were collected, and a whole-body dual-energy X-ray absorptiometry (DXA) scan performed within 2 weeks of birth. Follow up of the offspring occurred at regular intervals up to 6 years of age, including assessments of growth and bone health, as detailed in Table 1.

**Table 1.** Schedule of data collection in the MAVIDOS trial.

	Pregnancy			Birth	Childhood follow up				
	14 weeks	18–21 weeks	34 weeks	Birth	1 year	2 years	3 years	4 years	6–8 years
**Mother**									
Anthropometry	x		x	x					
Health, diet and lifestyle questionnaire	x		x						
Blood sampling	x		x						
Tablet count to assess compliance		x	x	x					
Foetal ultrasonography		x							
DXA				x				x	
pQCT								x	
**Father**									
Anthropometry	x^*^	x^*^	x^*^						
**Placenta**									
Placental and cord tissue collected				x					
**Offspring**									
Anthropometry				x	x	x	x	x	x
Health, diet and lifestyle questionnaire					x	x	x	x	x
DXA				x				x	x
pQCT								x	
HRpQCT									x
Hand-grip strength								x	x
Blood sampling				x (cord blood)				x	x

x denotes data collected at this time point.

* Measurement obtained once at one of these visits.

DXA, dual-energy X-ray absorptiometry; HRpQCT, high-resolution peripheral quantitative computed tomography; MAVIDOS, Maternal Vitamin D Osteoporosis Study; pQCT, peripheral quantitative computed tomography.

### Participant profile

A total of 1134 women were randomised into the study: 148 women were excluded pre-randomisation due to a screening of 25(O)H)D <25 nmol/L (*n* = 89) or >100 nmol/L (*n* = 59) and 965 (85.1%) remained in the study until delivery. These women were older, more likely of White ethnicity and better educated that those who withdrew. Over 94% of the women in the study were of White ethnicity. Just over 40% of the women were in their first pregnancy, 8% of the women smoked and nearly half were educated to degree level or higher. Of the 965 infants delivered into the study, 95% of infants were born at term; just over half were male. Compliance with the study medication, as assessed by tablet counts, was extremely high (>95%).

### Strengths and limitations

The double-blind placebo-controlled nature of the MAVIDOS study has generated the highest quality evidence with low risk of bias, and the large number of participants provided higher statistical power than some of the earlier much smaller studies of gestational vitamin D supplementation. The detailed phenotyping and comprehensive assessments of the women and offspring has enabled both the primary outcome and a number of other secondary hypotheses to be addressed.

The main limitation of the MAVIDOS trial was the exclusion of women with very low levels of 25(OH)D in early pregnancy. This was due to ethics considerations that these women should receive supplementation despite allowing all participants to take up to 400 IU/day vitamin D, as advised by the UK Department of Health,^21^ if they wished. As many observational studies have suggested that any detrimental effects of low vitamin D only become apparent at very low levels of 25(OH)D, women with 25(OH)D <25 nmol/L are potentially more likely to benefit from supplementation.^43–47^ This stipulation therefore reduced the ability to discern effects in the most vitamin D-deficient population.

Over 95% of the participants in the MAVIDOS trial were of White ethnicity. This reflects the local populations from which the women were recruited and does give more homogeneity to the study population, but also limits the generalisability of the study to women from other ethnic groups.

## Key findings of the MAVIDOS trial

### The effect of antenatal cholecalciferol supplementation on offspring bone mineralisation

**Figure 1.**
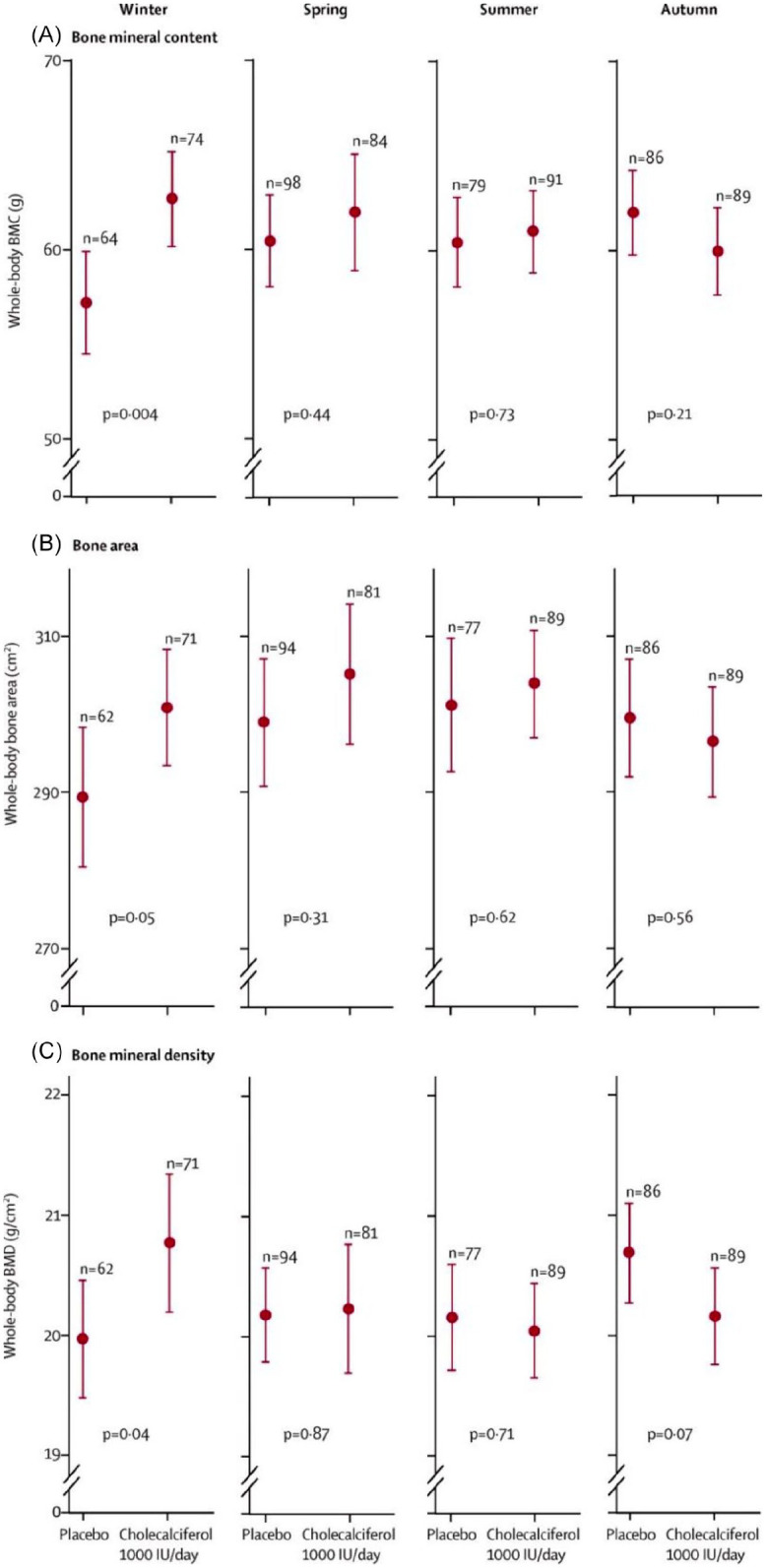
Neonatal whole-body BMC, bone area and BMD by intervention group and season of birth in the MAVIDOS trial. Data are shown as mean and 95% confidence interval. Winter is December to February, spring is March to May, summer is June to August and autumn is September to November. Reproduced with permission from Cooper *et al*.^9^ BMC, bone mineral content; BMD, bone mineral density; MAVIDOS, Maternal Vitamin D Osteoporosis Study.

The primary outcome of MAVIDOS was to determine the effect of antenatal cholecalciferol supplementation on offspring bone mass at birth. DXA scans of 736 infants were included in the analysis. The primary outcome of neonatal whole BMC did not differ statistically significantly between babies born to vitamin D-supplemented *versus* placebo mothers. However, a number of interactions had been prespecified within the original analysis plan.^42^ In consideration of the recognised seasonal variation in serum 25(OH)D, this included analysis of the effect by season of birth. Here, amongst winter/early spring deliveries when background 25(OH)D concentrations tend to be lowest in the population, and vitamin D supplementation prevented the fall in 25(OH)D from early to late pregnancy that was observed in the placebo group, the intervention led to a 0.5 standard deviation (SD) increase in neonatal whole body BMC compared with placebo, with no differences apparent in other seasons. A smaller, but significant interaction was also evident between season and areal BMD (aBMD) (Figure 1).^9^ To place the observed effect sizes into a clinical context, these are substantially larger than those observed in children with and without fracture, and therefore have the potential to be clinically important if sustained into later childhood. These findings would support the notion that the last trimester is the critical window for foetal bone mineral accretion, and although supplementation with 1000 IU/day did not result in the same achieved 25(OH)D level in women who delivered in winter compared with summer months, supplementation did prevent the decline in 25(OH)D status from early to late pregnancy observed in mothers who delivered in winter and were randomised to placebo. Interim follow up of Southampton children at 4 years has demonstrated a persisting benefit for WBLH BMC and aBMD, unstratified by season, with a further suggestion of interactions with childhood calcium intake and physical activity.^48^

This is consistent with recently published data from the Copenhagen Prospective Studies on Asthma in Childhood (COPSAC2010) trial.^49^ The Danish trial studied a higher dose of cholecalciferol supplementation (2800 IU/day *versus* 400 IU as control group), which was started later in gestation (24 weeks) but the observed differences in WBLH BMC and aBMD at 6 years of 0.15 and 0.2 SD, respectively, are of similar magnitude to the differences observed in MAVIDOS. The findings of MAVIDOS and COPSAC are in contrast to the findings of two small studies from India and Iran, which did not show a difference in infant bone mineralisation in response to antenatal cholecalciferol supplementation. However, major flaws in the methodology, substantial loss to follow up resulting in considerably smaller datasets (*n* = 52 and *n* = 25) and demographic differences between the randomisation groups limit the interpretation of these studies.^50,51^

Ongoing follow up of the MAVIDOS trial at 6 years of age will further assess the persistence of these findings, including analysis of both DXA and high-resolution peripheral quantitative computed tomography of the tibia.

### Mechanistic understanding of the effect of antenatal vitamin D supplementation and offspring bone mass

#### Epigenetic and biochemical mechanisms

**Figure 2.**
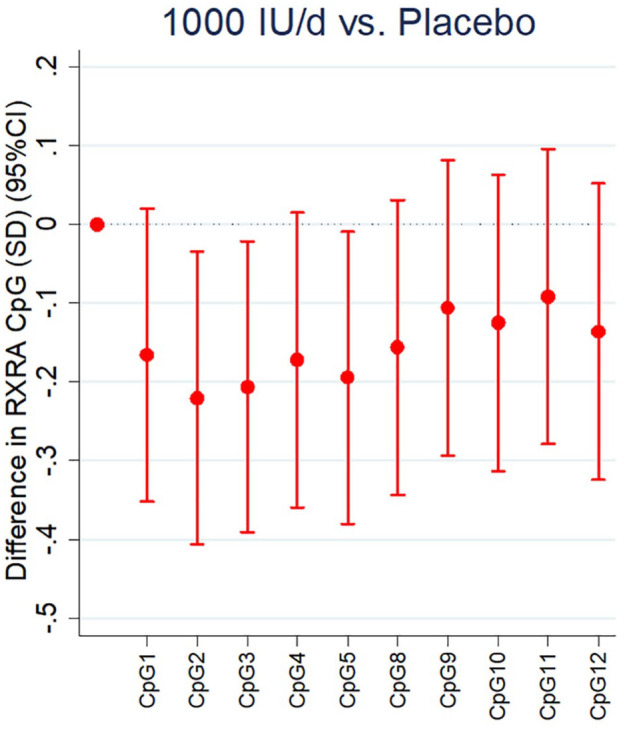
Effect of maternal vitamin D supplementation in pregnancy and perinatal umbilical cord methylation at CpG sites in the RXRA locus. Each bar comes from a separate linear regression. The outcomes are expressed in SDs. Reproduced with permission from Curtis *et al*.^59^ CI, confidence interval; RXRA, retinoid-X-receptor-A; SD, standard deviation.

It is becoming clear that some of the residual variance in BMD and fracture risk in adulthood might be explained by the influence of the environment on gene expression, both *in utero* and in early life.^52^ It is widely recognised that genes effectively provide a library of information that can be read (expressed) differently in different cells and tissues according to function and need, often in response to environmental cues.^53^ These effects are likely to be underpinned by epigenetic mechanisms, processes by which gene expression is modified but without changes in the DNA code itself. Epigenetic mechanisms include DNA methylation, histone modification and non-coding RNAs, the most widely studied of which is DNA methylation, the transfer of a methyl group to a particular genomic location, usually at the 5′ carbon position of cytosine adjacent to a guanine base, or CpG site. Methylation at regions of the genome particularly rich in CpG sites, for example, at the 5′ end of genes in regions known as CpG islands, often near to the promoter of a gene, may have important influences on that gene’s expression.^53–55^ Earlier analyses in the SWS birth cohort study showed perinatal DNA methylation at two loci of interest, *CDKN2A*, a key element in cell senescence^56^ and the retinoid-X-receptor-A (*RXRA*) gene, were inversely associated with childhood BMC corrected for body size at 4 years of age.^57,58^ RXRA forms a heterodimer with the vitamin D receptor and is essential in the nuclear action of 1,25(OH)_2_-vitamin D. Methylation at one CpG site was related to an estimate of free 25(OH)D. Using the MAVIDOS trial to establish the influence of maternal vitamin D supplementation on methylation of RXRA in a randomised controlled trial setting, it was demonstrated that supplementation with cholecalciferol in pregnancy is associated with reduced methylation at specific regions near to the *RXRA* promoter in foetal DNA derived from the umbilical cord of the offspring (Figure 2).^59^ This was in keeping with previous findings in the SWS, raising the possibility of site specificity for a molecular interaction between 25(OH)D in pregnancy and DNA methylation.^60^ Whilst the exact nature of the mechanistic underpinnings of these findings remains to be elucidated, there are several routes by which maternal 25(OH)D status might influence perinatal RXRA methylation. As RXRA forms a heterodimer with several nuclear hormones known to influence bone metabolism, including 1,25(OH)_2_-vitamin D, maternal 25(OH)D status may play a permissive role in the transcriptional regulation of the *RXRA* gene. Studies have shown that vitamin D may interact with the epigenome on multiple levels^61–65^ and evaluation of public data from Encyclopedia of DNA elements (ENCODE) suggested that methylation at the studied CpG sites is likely to have functional relevance.

The MAVIDOS trial also offered the opportunity to study the impact of gestational vitamin D supplementation on the maternal skeleton, as maternal calcium homeostasis adapts to meet the calcium demands of the developing foetus,^66–68^ though its response to vitamin D supplementation is not well defined. Biochemical markers of bone turnover offer a noninvasive method of monitoring changes in bone resorption or formation during pregnancy;^69^ in the MAVIDOS trial maternal urinary C-terminal telopeptide of type I collagen (CTX) was measured at 14 weeks and 34 weeks gestation. At the population level, there is evidence of inverse associations between 25(OH)D concentrations and markers of bone resorption such as CTX.^70–73^ In the MAVIDOS cohort maternal gestational cholecalciferol supplementation was associated with a smaller gestational increase in bone resorption markers compared with placebo. Whilst maternal urinary CTX almost doubled from 14 weeks’ to 34 weeks’ gestation in both randomisation groups, the conditional increase in CTX from early to late pregnancy was lower in the cholecalciferol-supplemented group compared with the placebo group. Furthermore, late pregnancy CTX was inversely associated with postpartum measures of maternal bone from DXA.^74^ These findings are consistent with a protective effect of gestational vitamin D supplementation on maternal bone health, however, long-term follow up of both mothers and offspring, with repeat assessments of bone indices, is needed.

#### The effect of cholecalciferol supplementation on vitamin D status and determinants of the response to supplementation

**Figure 3.**
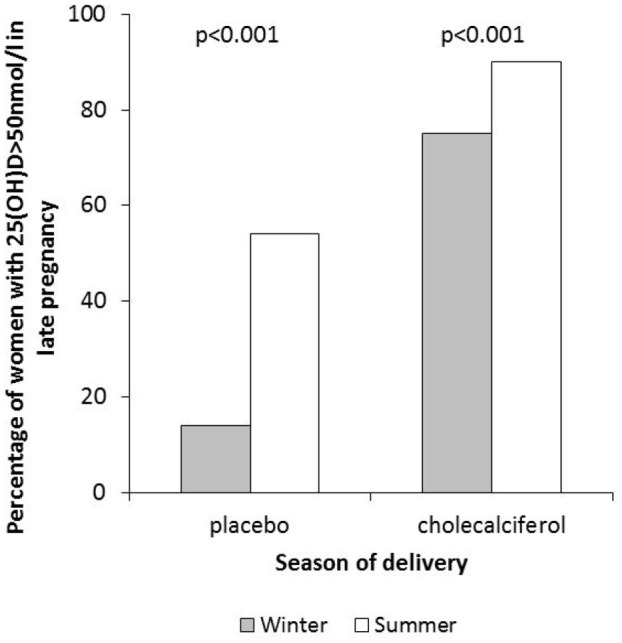
Proportion of women achieving vitamin D replete status (25(OH)D >50 nmol/L) in late pregnancy stratified by randomisation to placebo or 1000 IU/day cholecalciferol and season of delivery. Winter was defined as December to May.

The MAVIDOS trial has aided understanding of the biochemical response to supplementation. We clearly demonstrated that antenatal supplementation with 1000 IU/day increased maternal 25(OH)D status in late pregnancy; 83% of women randomised to cholecalciferol achieved a 25(OH)D >50 nmol/L at 34 weeks’ gestation compared with only 36% in the placebo group (Figure 3). No participant reported symptoms suggestive of vitamin D toxicity,^75^ although women with a baseline 25(OH)D >100 nmol/L, who might have been at higher risk of toxicity were excluded from participation. Nonetheless, this finding supports other studies that have also demonstrated a rise in biochemical vitamin D status in response to pregnancy supplementation, and even higher doses up to 4000 IU/day have not been associated with adverse clinical outcomes in other trials.^76^

Importantly, despite high levels of compliance in this group, 17% of women did not achieve vitamin D repletion in late pregnancy, suggesting that perhaps higher doses are required to achieve this (Figure 3). However, interestingly, a previous study in New Zealand did not show a difference in repletion rates (25(OH)D >50 nmol/L) for doses of 1000 or 2000 IU/day cholecalciferol during pregnancy.^77^ Indeed, in the MAVIDOS trial evidence for a ceiling effect of supplementation was also present. Thus, there was a smaller difference in 25(OH)D concentrations at 34 weeks gestation between the placebo and cholecalciferol groups with increasing baseline 25(OH)D. This interaction between baseline 25(OH)D and randomisation group on achieved 25(OH)D was highly statistically significant (*p* < 0.001).

Using the MAVIDOS trial we identified a number of factors that were associated with the 25(OH)D response to cholecalciferol supplementation. Firstly, it is clear that the seasonal variation in 25(OH)D status at latitudes far from the equator^78^ is not abolished by this level of antenatal supplementation at latitudes within the UK^75^ (Figure 3). In addition, using multivariate analysis, compliance and baseline 25(OH)D were positively associated with the achieved 25(OH)D in late pregnancy following antenatal vitamin D supplementation, whereas weight gain during pregnancy was negatively associated.^75^ Similarly, Black and Minority Ethnic ethnicity was associated with a higher risk of not achieving vitamin D replete status in the women with supplementation, consistent with the finding of Hollis *et al*.^76^ that even with 4000 IU/day vitamin D during pregnancy, African-American women had lower 25(OH)D in late pregnancy than White or Hispanic women. Maternal weight gain during pregnancy was negatively associated with the response to cholecalciferol supplementation in the MAVIDOS trial. This finding is consistent with our earlier observation using data from the SWS birth cohort study, which showed greater gestational weight gain was negatively associated with the tracking of 25(OH)D from early to late pregnancy independent of supplement use.^78^ Other studies in nonpregnant adults have similarly demonstrated that over 50% of the variance in 25(OH)D increment in response to supplementation is explained by body weight.^79^

Genetic variation in the response to cholecalciferol was also identified in the MAVIDOS cohort. A number of single nucleotide polymorphisms (SNPs) within the vitamin D metabolism pathway, including in genes encoding 7-dehydrocholesterol reductase in the skin, 25-hydroxylase, 24-hydroxylase and vitamin D binding protein (DBP) have been identified as significantly associated with 25(OH)D status in nonpregnant populations.^80,81^ In women of White ethnicity in the MAVIDOS trial, SNPs in genes encoding 25-hydroxylase (CYP2R1) and DBP (GC) were associated with the achieved 25(OH)D after supplementation.^82^

**Figure 4.**
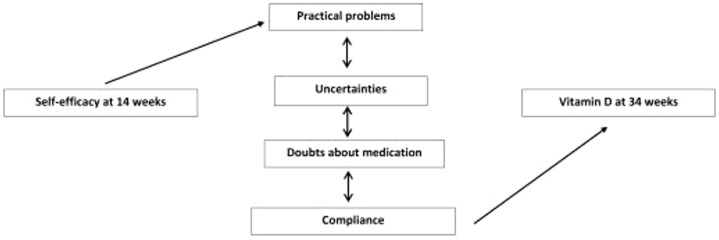
Conceptual model summarising the relationships between self-efficacy, vitamin D at 34 weeks, compliance with trial protocol, practical problems taking the study medication, uncertainty and doubts about taking the medication. Reproduced with permission from Barker *et al*.^83^

A substudy of the MAVIDOS trial assessed psychological characteristics associated with compliance with the study medication, including self-efficacy, defined as the belief that one is capable of carrying out a specific behaviour. Women with higher self-efficacy experienced fewer practical problems with taking the supplement. Experiencing practical problems, having doubts and uncertainties about the medication and rate of compliance were all strongly associated with one another (*p* < 0.05 for all), and the latter with 25(OH)D achieved postsupplementation (Figure 4).^83^

## Implications for clinical practice

Guidelines in the UK and USA recommend pregnant women should take 400–600 IU/day cholecalciferol.^21,84^ The findings of the MAVIDOS trial^42,48^ and COPSAC2010^49^ suggest that higher doses of antenatal vitamin D supplementation have beneficial effects on offspring skeletal mineralisation. Attempts to replicate these findings in the Southampton Pregnancy Intervention for the Next Generation (SPRING) study is currently in progress,^85^ and ongoing follow up of the MAVIDOS trial at 6–8 years aims to demonstrate persistence of the observed effect further into childhood.

It is clear from the MAVIDOS trial that 1000 IU/day cholecalciferol does not abolish the seasonal variation in 25(OH)D status during late pregnancy and that a large proportion of women who were supplemented with this dose, and in particular those who delivered in winter months, will still have a 25(OH)D level in late pregnancy of <50 nmol/L. As such, if the aim of supplementation is to increase maternal 25(OH)D to >50 nmol/L, which is often considered the definition for repletion, then it is likely that 400 IU/day will not achieve this in many women. However, a change in public-health policy needs to be based on established benefits in high-quality randomised controlled trials, and whilst the findings of the MAVIDOS trial begin to demonstrate important clinical outcomes, consistent findings across more randomised controlled trials are required.

It is also important to be certain that in addition to benefits a higher dose will not be harmful. The literature with regards to falls risk in older individuals suggests that moderate doses of vitamin D (600–1000 IU/day) may have a beneficial effect whilst high bolus doses increase the risk of falls.^86^ There were no obvious side effects of 1000 IU/day during pregnancy in the MAVIDOS trial or up to 4000 IU/day in another pregnancy study,^76^ and there appears to be a ceiling effect to the achievable 25(OH)D following this level of supplementation when baseline 25(OH)D levels are high. However, a clear benefit of higher dose antenatal supplementation needs to be demonstrated before it can be recommended in routine clinical practice.

The MAVIDOS trial has confirmed that a number of maternal factors are associated with poorer biochemical response to supplementation, including compliance, low baseline 25(OH)D, non-White ethnicity and weight gain. In clinical practice, counselling women on the risk of vitamin D deficiency and need for supplementation is vital, particularly those with well-recognised risk factors for deficiency. This should be routinely reviewed at every antenatal appointment. In the UK, serum 25(OH)D is not routinely assessed in early pregnancy, and the additional economic cost of this might be difficult to justify in light of the low likelihood of harm from low-dose cholecalciferol supplementation. It perhaps needs to be demonstrated in research studies that dosing schedules based on baseline 25(OH)D will achieve higher vitamin D repletion in a greater number of women and improved clinical outcomes before measurement of 25(OH)D in early pregnancy could be deemed necessary. Similarly, it is clear that the degree of weight gain during pregnancy is associated with 25(OH)D status and the response to supplementation. Women with higher than recommended weight gain should be counselled on the need for vitamin D supplementation to maintain their 25(OH)D status.

## Conclusion

The findings from the MAVIDOS trial of potential beneficial effects of maternal vitamin D supplementation during pregnancy on offspring bone mass, and the elucidation of possible underlying mechanisms, have increased our understanding of the role of vitamin D in pregnancy. Results from the MAVIDOS trial have informed policy from bodies such as the UK Scientific Advisory Committee on Nutrition^87^ and the National Institute for Health Research.^88^ These findings, and those from ongoing follow up, data analysis and substudies within the MAVIDOS trial, together with further independent trials such as SPRING, will be critical to future public-health advice on vitamin D supplementation in pregnancy.
